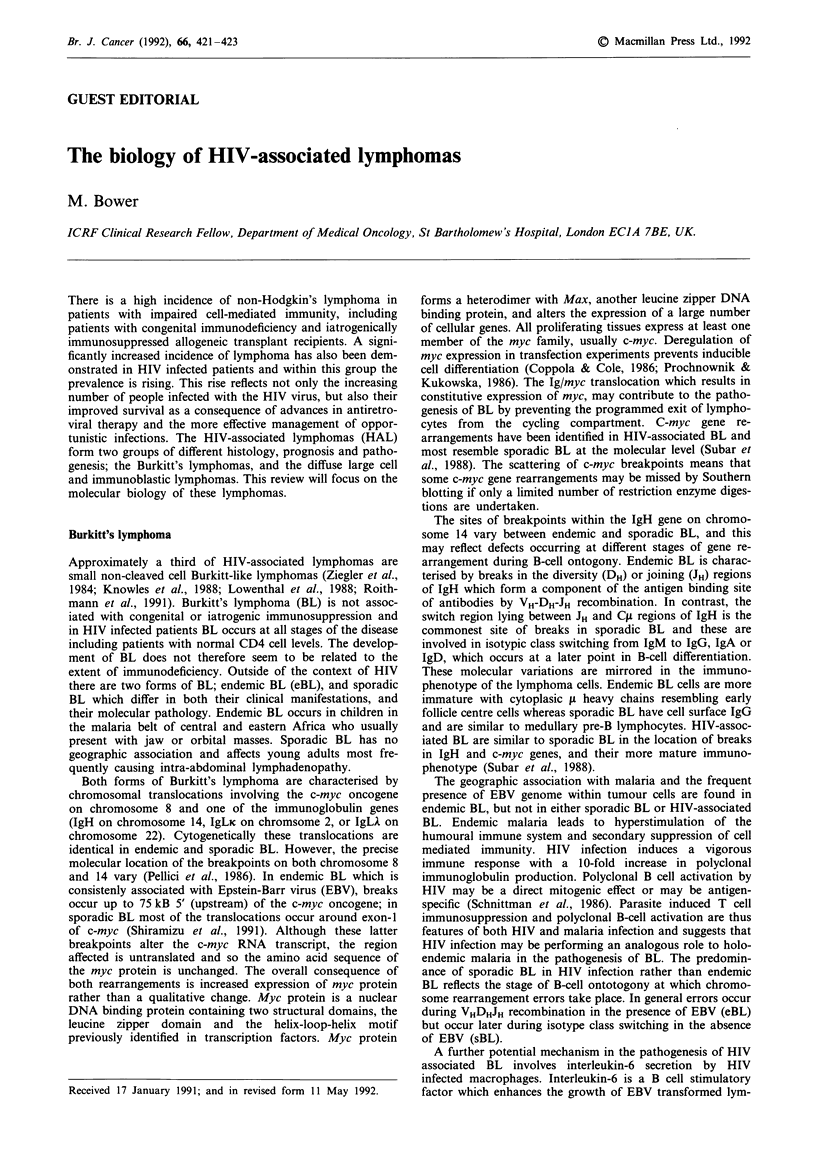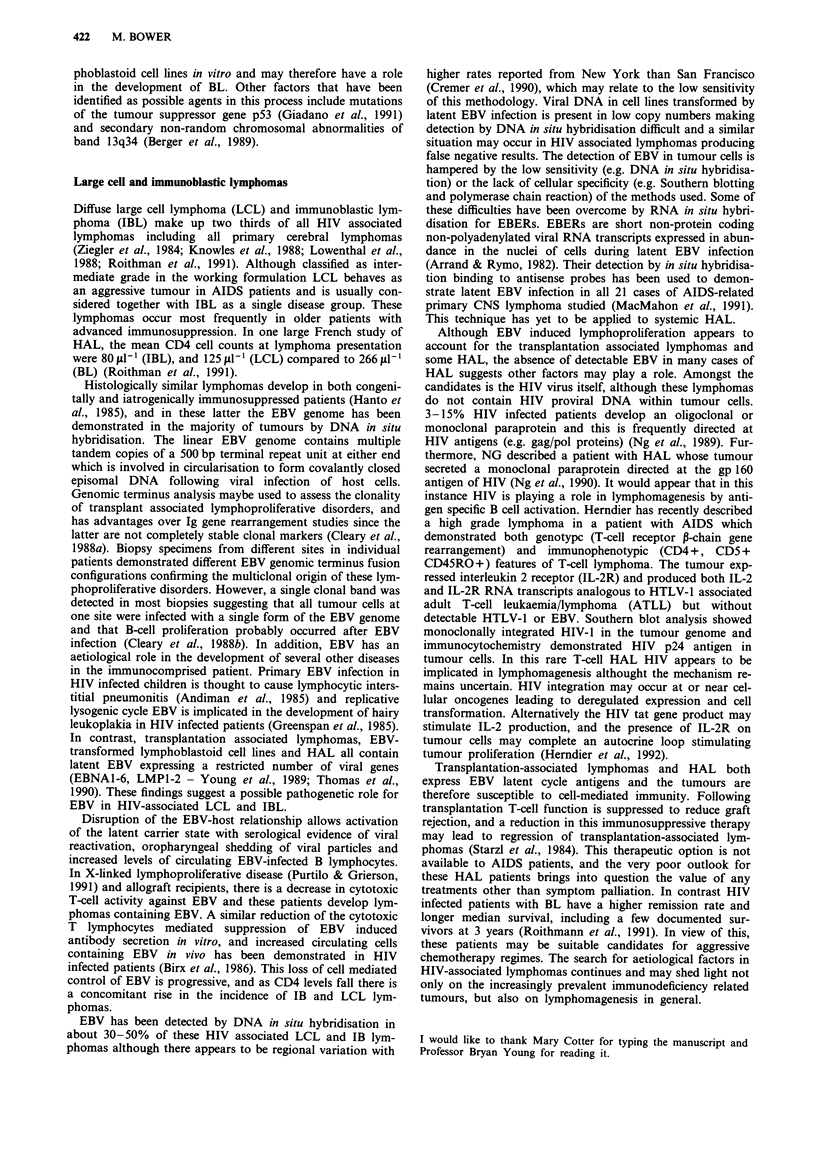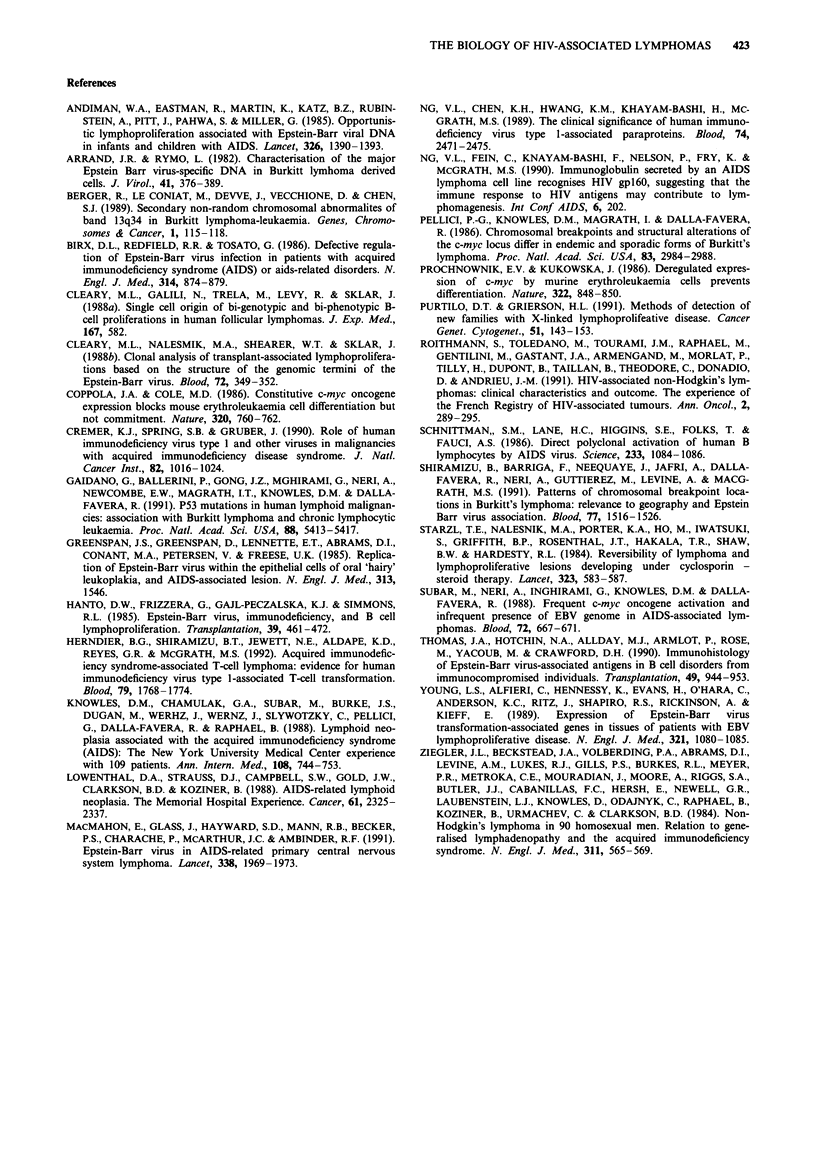# The biology of HIV-associated lymphomas.

**DOI:** 10.1038/bjc.1992.289

**Published:** 1992-09

**Authors:** M. Bower


Br. J. Cancer (1992), 66, 421–423 © Macmillan Press Ltd., 1992

GUEST EDITORIAL

### The biology of HIV-associated lymphomas

M. Bower

*ICRF Clinical Research Fellow, Department of Medical Oncology, St Bartholomew's Hospital, London EC1A 7BE, UK.*

There is a high incidence of non-Hodgkin's lymphoma in patients with impaired cell-mediated immunity, including patients with congenital immunodeficiency and iatrogenically immunosuppressed allogeneic transplant recipients. A significantly increased incidence of lymphoma has also been demonstrated in HIV infected patients and within this group the prevalence is rising. This rise reflects not only the increasing number of people infected with the HIV virus, but also their improved survival as a consequence of advances in antiretroviral therapy and the more effective management of opportunistic infections. The HIV-associated lymphomas (HAL) form two groups of different histology, prognosis and pathogenesis; the Burkitt's lymphomas, and the diffuse large cell and immunoblastic lymphomas. This review will focus on the molecular biology of these lymphomas.

### Burkitt's lymphoma

Approximately a third of HIV-associated lymphomas are small non-cleaved cell Burkitt-like lymphomas (Ziegler *et al.*, 1984; Knowles *et al.*, 1988; Lowenthal *et al.*, 1988; Roithmann *et al.*, 1991). Burkitt's lymphoma (BL) is not associated with congenital or iatrogenic immunosuppression and in HIV infected patients BL occurs at all stages of the disease including patients with normal CD4 cell levels. The development of BL does not therefore seem to be related to the extent of immunodeficiency. Outside of the context of HIV there are two forms of BL; endemic BL (eBL), and sporadic BL which differ in both their clinical manifestations, and their molecular pathology. Endemic BL occurs in children in the malaria belt of central and eastern Africa who usually present with jaw or orbital masses. Sporadic BL has no geographic association and affects young adults most frequently causing intra-abdominal lymphadenopathy.

Both forms of Burkitt's lymphoma are characterised by chromosomal translocations involving the c-*myc* oncogene on chromosome 8 and one of the immunoglobulin genes (IgH on chromosome 14, IgLκ on chromsome 2, or IgLλ on chromosome 22). Cytogenetically these translocations are identical in endemic and sporadic BL. However, the precise molecular location of the breakpoints on both chromosome 8 and 14 vary (Pellici *et al.*, 1986). In endemic BL which is consistenly associated with Epstein-Barr virus (EBV), breaks occur up to 75 kB 5′ (upstream) of the c-*myc* oncogene; in sporadic BL most of the translocations occur around exon-1 of c-*myc* (Shiramizu *et al.*, 1991). Although these latter breakpoints alter the c-*myc* RNA transcript, the region affected is untranslated and so the amino acid sequence of the *myc* protein is unchanged. The overall consequence of both rearrangements is increased expression of *myc* protein rather than a qualitative change. *Myc* protein is a nuclear DNA binding protein containing two structural domains, the leucine zipper domain and the helix-loop-helix motif previously identified in transcription factors. *Myc* protein forms a heterodimer with *Max*, another leucine zipper DNA binding protein, and alters the expression of a large number of cellular genes. All proliferating tissues express at least one member of the *myc* family, usually c-*myc*. Deregulation of *myc* expression in transfection experiments prevents inducible cell differentiation (Coppola & Cole, 1986; Prochnownik & Kukowska, 1986). The Ig/*myc* translocation which results in constitutive expression of *myc*, may contribute to the pathogenesis of BL by preventing the programmed exit of lymphocytes from the cycling compartment. C-*myc* gene rearrangements have been identified in HIV-associated BL and most resemble sporadic BL at the molecular level (Subar *et al.*, 1988). The scattering of c-*myc* breakpoints means that some c-*myc* gene rearrangements may be missed by Southern blotting if only a limited number of restriction enzyme digestions are undertaken.

The sites of breakpoints within the IgH gene on chromosome 14 vary between endemic and sporadic BL, and this may reflect defects occurring at different stages of gene rearrangement during B-cell ontogony. Endemic BL is characterised by breaks in the diversity ($D_H$) or joining ($J_H$) regions of IgH which form a component of the antigen binding site of antibodies by $V_H$-$D_H$-$J_H$ recombination. In contrast, the switch region lying between $J_H$ and $C\mu$ regions of IgH is the commonest site of breaks in sporadic BL and these are involved in isotypic class switching from IgM to IgG, IgA or IgD, which occurs at a later point in B-cell differentiation. These molecular variations are mirrored in the immunophenotype of the lymphoma cells. Endemic BL cells are more immature with cytoplasic $\mu$ heavy chains resembling early follicle centre cells whereas sporadic BL have cell surface IgG and are similar to medullary pre-B lymphocytes. HIV-associated BL are similar to sporadic BL in the location of breaks in IgH and c-*myc* genes, and their more mature immunophenotype (Subar *et al.*, 1988).

The geographic association with malaria and the frequent presence of EBV genome within tumour cells are found in endemic BL, but not in either sporadic BL or HIV-associated BL. Endemic malaria leads to hyperstimulation of the humoural immune system and secondary suppression of cell mediated immunity. HIV infection induces a vigorous immune response with a 10-fold increase in polyclonal immunoglobulin production. Polyclonal B cell activation by HIV may be a direct mitogenic effect or may be antigen-specific (Schnittman *et al.*, 1986). Parasite induced T cell immunosuppression and polyclonal B-cell activation are thus features of both HIV and malaria infection and suggests that HIV infection may be performing an analogous role to holoendemic malaria in the pathogenesis of BL. The predominance of sporadic BL in HIV infection rather than endemic BL reflects the stage of B-cell ontotogony at which chromosome rearrangement errors take place. In general errors occur during $V_HD_HJ_H$ recombination in the presence of EBV (eBL) but occur later during isotype class switching in the absence of EBV (sBL).

A further potential mechanism in the pathogenesis of HIV associated BL involves interleukin-6 secretion by HIV infected macrophages. Interleukin-6 is a B cell stimulatory factor which enhances the growth of EBV transformed lym-

Received 17 January 1991; and in revised form 11 May 1992.

phoblastoid cell lines *in vitro* and may therefore have a role in the development of BL. Other factors that have been identified as possible agents in this process include mutations of the tumour suppressor gene p53 (Giadano *et al.*, 1991) and secondary non-random chromosomal abnormalities of band 13q34 (Berger *et al.*, 1989).

**Large cell and immunoblastic lymphomas**

Diffuse large cell lymphoma (LCL) and immunoblastic lymphoma (IBL) make up two thirds of all HIV associated lymphomas including all primary cerebral lymphomas (Ziegler *et al.*, 1984; Knowles *et al.*, 1988; Lowenthal *et al.*, 1988; Roithman *et al.*, 1991). Although classified as intermediate grade in the working formulation LCL behaves as an aggressive tumour in AIDS patients and is usually considered together with IBL as a single disease group. These lymphomas occur most frequently in older patients with advanced immunosuppression. In one large French study of HAL, the mean CD4 cell counts at lymphoma presentation were 80 $\mu l^{-1}$ (IBL), and 125 $\mu l^{-1}$ (LCL) compared to 266 $\mu l^{-1}$ (BL) (Roithman *et al.*, 1991).

Histologically similar lymphomas develop in both congenitally and iatrogenically immunosuppressed patients (Hanto *et al.*, 1985), and in these latter the EBV genome has been demonstrated in the majority of tumours by DNA *in situ* hybridisation. The linear EBV genome contains multiple tandem copies of a 500 bp terminal repeat unit at either end which is involved in circularisation to form covalantly closed episomal DNA following viral infection of host cells. Genomic terminus analysis maybe used to assess the clonality of transplant associated lymphoproliferative disorders, and has advantages over Ig gene rearrangement studies since the latter are not completely stable clonal markers (Cleary *et al.*, 1988*a*). Biopsy specimens from different sites in individual patients demonstrated different EBV genomic terminus fusion configurations confirming the multiclonal origin of these lymphoproliferative disorders. However, a single clonal band was detected in most biopsies suggesting that all tumour cells at one site were infected with a single form of the EBV genome and that B-cell proliferation probably occurred after EBV infection (Cleary *et al.*, 1988*b*). In addition, EBV has an aetiological role in the development of several other diseases in the immunocomprised patient. Primary EBV infection in HIV infected children is thought to cause lymphocytic interstitial pneumonitis (Andiman *et al.*, 1985) and replicative lysogenic cycle EBV is implicated in the development of hairy leukoplakia in HIV infected patients (Greenspan *et al.*, 1985). In contrast, transplantation associated lymphomas, EBV-transformed lymphoblastoid cell lines and HAL all contain latent EBV expressing a restricted number of viral genes (EBNA1-6, LMP1-2 - Young *et al.*, 1989; Thomas *et al.*, 1990). These findings suggest a possible pathogenetic role for EBV in HIV-associated LCL and IBL.

Disruption of the EBV-host relationship allows activation of the latent carrier state with serological evidence of viral reactivation, oropharyngeal shedding of viral particles and increased levels of circulating EBV-infected B lymphocytes. In X-linked lymphoproliferative disease (Purtilo & Grierson, 1991) and allograft recipients, there is a decrease in cytotoxic T-cell activity against EBV and these patients develop lymphomas containing EBV. A similar reduction of the cytotoxic T lymphocytes mediated suppression of EBV induced antibody secretion *in vitro*, and increased circulating cells containing EBV *in vivo* has been demonstrated in HIV infected patients (Birx *et al.*, 1986). This loss of cell mediated control of EBV is progressive, and as CD4 levels fall there is a concomitant rise in the incidence of IB and LCL lymphomas.

EBV has been detected by DNA *in situ* hybridisation in about 30–50% of these HIV associated LCL and IB lymphomas although there appears to be regional variation with higher rates reported from New York than San Francisco (Cremer *et al.*, 1990), which may relate to the low sensitivity of this methodology. Viral DNA in cell lines transformed by latent EBV infection is present in low copy numbers making detection by DNA *in situ* hybridisation difficult and a similar situation may occur in HIV associated lymphomas producing false negative results. The detection of EBV in tumour cells is hampered by the low sensitivity (e.g. DNA *in situ* hybridisation) or the lack of cellular specificity (e.g. Southern blotting and polymerase chain reaction) of the methods used. Some of these difficulties have been overcome by RNA *in situ* hybridisation for EBERs. EBERs are short non-protein coding non-polyadenylated viral RNA transcripts expressed in abundance in the nuclei of cells during latent EBV infection (Arrand & Rymo, 1982). Their detection by *in situ* hybridisation binding to antisense probes has been used to demonstrate latent EBV infection in all 21 cases of AIDS-related primary CNS lymphoma studied (MacMahon *et al.*, 1991). This technique has yet to be applied to systemic HAL.

Although EBV induced lymphoproliferation appears to account for the transplantation associated lymphomas and some HAL, the absence of detectable EBV in many cases of HAL suggests other factors may play a role. Amongst the candidates is the HIV virus itself, although these lymphomas do not contain HIV proviral DNA within tumour cells. 3–15% HIV infected patients develop an oligoclonal or monoclonal paraprotein and this is frequently directed at HIV antigens (e.g. gag/pol proteins) (Ng *et al.*, 1989). Furthermore, NG described a patient with HAL whose tumour secreted a monoclonal paraprotein directed at the gp 160 antigen of HIV (Ng *et al.*, 1990). It would appear that in this instance HIV is playing a role in lymphomagenesis by antigen specific B cell activation. Herndier has recently described a high grade lymphoma in a patient with AIDS which demonstrated both genotypc (T-cell receptor $\beta$-chain gene rearrangement) and immunophenotypic (CD4+, CD5+ CD45RO+) features of T-cell lymphoma. The tumour expressed interleukin 2 receptor (IL-2R) and produced both IL-2 and IL-2R RNA transcripts analogous to HTLV-1 associated adult T-cell leukaemia/lymphoma (ATLL) but without detectable HTLV-1 or EBV. Southern blot analysis showed monoclonally integrated HIV-1 in the tumour genome and immunocytochemistry demonstrated HIV p24 antigen in tumour cells. In this rare T-cell HAL HIV appears to be implicated in lymphomagenesis althought the mechanism remains uncertain. HIV integration may occur at or near cellular oncogenes leading to deregulated expression and cell transformation. Alternatively the HIV tat gene product may stimulate IL-2 production, and the presence of IL-2R on tumour cells may complete an autocrine loop stimulating tumour proliferation (Herndier *et al.*, 1992).

Transplantation-associated lymphomas and HAL both express EBV latent cycle antigens and the tumours are therefore susceptible to cell-mediated immunity. Following transplantation T-cell function is suppressed to reduce graft rejection, and a reduction in this immunosuppressive therapy may lead to regression of transplantation-associated lymphomas (Starzl *et al.*, 1984). This therapeutic option is not available to AIDS patients, and the very poor outlook for these HAL patients brings into question the value of any treatments other than symptom palliation. In contrast HIV infected patients with BL have a higher remission rate and longer median survival, including a few documented survivors at 3 years (Roithmann *et al.*, 1991). In view of this, these patients may be suitable candidates for aggressive chemotherapy regimes. The search for aetiological factors in HIV-associated lymphomas continues and may shed light not only on the increasingly prevalent immunodeficiency related tumours, but also on lymphomagenesis in general.

I would like to thank Mary Cotter for typing the manuscript and Professor Bryan Young for reading it.

### References

ANDIMAN, W.A., EASTMAN, R., MARTIN, K., KATZ, B.Z., RUBINSTEIN, A., PITT, J., PAHWA, S. & MILLER, G. (1985). Opportunistic lymphoproliferation associated with Epstein-Barr viral DNA in infants and children with AIDS. *Lancet*, **326**, 1390–1393.

ARRAND, J.R. & RYMO, L. (1982). Characterisation of the major Epstein Barr virus-specific DNA in Burkitt lymhoma derived cells. *J. Virol.*, **41**, 376–389.

BERGER, R., LE CONIAT, M., DEVVE, J., VECCHIONE, D. & CHEN, S.J. (1989). Secondary non-random chromosomal abnormalites of band 13q34 in Burkitt lymphoma-leukaemia. *Genes, Chromosomes & Cancer*, **1**, 115–118.

BIRX, D.L., REDFIELD, R.R. & TOSATO, G. (1986). Defective regulation of Epstein-Barr virus infection in patients with acquired immunodeficiency syndrome (AIDS) or aids-related disorders. *N. Engl. J. Med.*, **314**, 874–879.

CLEARY, M.L., GALILI, N., TRELA, M., LEVY, R. & SKLAR, J. (1988*a*). Single cell origin of bi-genotypic and bi-phenotypic B-cell proliferations in human follicular lymphomas. *J. Exp. Med.*, **167**, 582.

CLEARY, M.L., NALESMIK, M.A., SHEARER, W.T. & SKLAR, J. (1988*b*). Clonal analysis of transplant-associated lymphoproliferations based on the structure of the genomic termini of the Epstein-Barr virus. *Blood*, **72**, 349–352.

COPPOLA, J.A. & COLE, M.D. (1986). Constitutive c-*myc* oncogene expression blocks mouse erythroleukaemia cell differentiation but not commitment. *Nature*, **320**, 760–762.

CREMER, K.J., SPRING, S.B. & GRUBER, J. (1990). Role of human immunodeficiency virus type 1 and other viruses in malignancies with acquired immunodeficiency disease syndrome. *J. Natl. Cancer Inst.*, **82**, 1016–1024.

GAIDANO, G., BALLERINI, P., GONG, J.Z., MGHIRAMI, G., NERI, A., NEWCOMBE, E.W., MAGRATH, I.T., KNOWLES, D.M. & DALLA-FAVERA, R. (1991). P53 mutations in human lymphoid malignancies: association with Burkitt lymphoma and chronic lymphocytic leukaemia. *Proc. Natl. Acad. Sci. USA*, **88**, 5413–5417.

GREENSPAN, J.S., GREENSPAN, D., LENNETTE, E.T., ABRAMS, D.I., CONANT, M.A., PETERSEN, V. & FREESE, U.K. (1985). Replication of Epstein-Barr virus within the epithelial cells of oral 'hairy' leukoplakia, and AIDS-associated lesion. *N. Engl. J. Med.*, **313**, 1546.

HANTO, D.W., FRIZZERA, G., GAJL-PECZALSKA, K.J. & SIMMONS, R.L. (1985). Epstein-Barr virus, immunodeficiency, and B cell lymphoproliferation. *Transplantation*, **39**, 461–472.

HERNDIER, B.G., SHIRAMIZU, B.T., JEWETT, N.E., ALDAPE, K.D., REYES, G.R. & MCGRATH, M.S. (1992). Acquired immunodeficiency syndrome-associated T-cell lymphoma: evidence for human immunodeficiency virus type 1-associated T-cell transformation. *Blood*, **79**, 1768–1774.

KNOWLES, D.M., CHAMULAK, G.A., SUBAR, M., BURKE, J.S., DUGAN, M., WERHZ, J., WERNZ, J., SLYWOTZKY, C., PELLICI, G., DALLA-FAVERA, R. & RAPHAEL, B. (1988). Lymphoid neoplasia associated with the acquired immunodeficiency syndrome (AIDS): The New York University Medical Center experience with 109 patients. *Ann. Intern. Med.*, **108**, 744–753.

LOWENTHAL, D.A., STRAUSS, D.J., CAMPBELL, S.W., GOLD, J.W., CLARKSON, B.D. & KOZINER, B. (1988). AIDS-related lymphoid neoplasia. The Memorial Hospital Experience. *Cancer*, **61**, 2325–2337.

MACMAHON, E., GLASS, J., HAYWARD, S.D., MANN, R.B., BECKER, P.S., CHARACHE, P., MCARTHUR, J.C. & AMBINDER, R.F. (1991). Epstein-Barr virus in AIDS-related primary central nervous system lymphoma. *Lancet*, **338**, 1969–1973.

NG, V.L., CHEN, K.H., HWANG, K.M., KHAYAM-BASHI, H., MCGRATH, M.S. (1989). The clinical significance of human immunodeficiency virus type 1-associated paraproteins. *Blood*, **74**, 2471–2475.

NG, V.L., FEIN, C., KNAYAM-BASHI, F., NELSON, P., FRY, K. & MCGRATH, M.S. (1990). Immunoglobulin secreted by an AIDS lymphoma cell line recognises HIV gp160, suggesting that the immune response to HIV antigens may contribute to lymphomagenesis. *Int Conf AIDS*, **6**, 202.

PELLICI, P.-G., KNOWLES, D.M., MAGRATH, I. & DALLA-FAVERA, R. (1986). Chromosomal breakpoints and structural alterations of the c-*myc* locus differ in endemic and sporadic forms of Burkitt's lymphoma. *Proc. Natl. Acad. Sci. USA*, **83**, 2984–2988.

PROCHNOWNIK, E.V. & KUKOWSKA, J. (1986). Deregulated expression of c-*myc* by murine erythroleukaemia cells prevents differentiation. *Nature*, **322**, 848–850.

PURTILO, D.T. & GRIERSON, H.L. (1991). Methods of detection of new families with X-linked lymphoprolifeative disease. *Cancer Genet. Cytogenet.*, **51**, 143–153.

ROITHMANN, S., TOLEDANO, M., TOURAMI, J.M., RAPHAEL, M., GENTILINI, M., GASTANT, J.A., ARMENGAND, M., MORLAT, P., TILLY, H., DUPONT, B., TAILLAN, B., THEODORE, C., DONADIO, D. & ANDRIEU, J.-M. (1991). HIV-associated non-Hodgkin's lymphomas: clinical characteristics and outcome. The experience of the French Registry of HIV-associated tumours. *Ann. Oncol.*, **2**, 289–295.

SCHNITTMAN,, S.M., LANE, H.C., HIGGINS, S.E., FOLKS, T. & FAUCI, A.S. (1986). Direct polyclonal activation of human B lymphocytes by AIDS virus. *Science*, **233**, 1084–1086.

SHIRAMIZU, B., BARRIGA, F., NEEQUAYE, J., JAFRI, A., DALLA-FAVERA, R., NERI, A., GUTTIEREZ, M., LEVINE, A. & MACGRATH, M.S. (1991). Patterns of chromosomal breakpoint locations in Burkitt's lymphoma: relevance to geography and Epstein Barr virus association. *Blood*, **77**, 1516–1526.

STARZL, T.E., NALESNIK, M.A., PORTER, K.A., HO, M., IWATSUKI, S., GRIFFITH, B.P., ROSENTHAL, J.T., HAKALA, T.R., SHAW, B.W. & HARDESTY, R.L. (1984). Reversibility of lymphoma and lymphoproliferative lesions developing under cyclosporin – steroid therapy. *Lancet*, **323**, 583–587.

SUBAR, M., NERI, A., INGHIRAMI, G., KNOWLES, D.M. & DALLA-FAVERA, R. (1988). Frequent c-*myc* oncogene activation and infrequent presence of EBV genome in AIDS-associated lymphomas. *Blood*, **72**, 667–671.

THOMAS, J.A., HOTCHIN, N.A., ALLDAY, M.J., ARMLOT, P., ROSE, M., YACOUB, M. & CRAWFORD, D.H. (1990). Immunohistology of Epstein-Barr virus-associated antigens in B cell disorders from immunocompromised individuals. *Transplantation*, **49**, 944–953.

YOUNG, L.S., ALFIERI, C., HENNESSY, K., EVANS, H., O'HARA, C., ANDERSON, K.C., RITZ, J., SHAPIRO, R.S., RICKINSON, A. & KIEFF, E. (1989). Expression of Epstein-Barr virus transformation-associated genes in tissues of patients with EBV lymphoproliferative disease. *N. Engl. J. Med.*, **321**, 1080–1085.

ZIEGLER, J.L., BECKSTEAD, J.A., VOLBERDING, P.A., ABRAMS, D.I., LEVINE, A.M., LUKES, R.J., GILLS, P.S., BURKES, R.L., MEYER, P.R., METROKA, C.E., MOURADIAN, J., MOORE, A., RIGGS, S.A., BUTLER, J.J., CABANILLAS, F.C., HERSH, E., NEWELL, G.R., LAUBENSTEIN, L.J., KNOWLES, D., ODAJNYK, C., RAPHAEL, B., KOZINER, B., URMACHEV, C. & CLARKSON, B.D. (1984). Non-Hodgkin's lymphoma in 90 homosexual men. Relation to generalised lymphadenopathy and the acquired immunodeficiency syndrome. *N. Engl. J. Med.*, **311**, 565–569.